# The Potential of *Campanula takesimana* Callus Extract to Enhance Skin Barrier Function

**DOI:** 10.3390/ijms242417333

**Published:** 2023-12-11

**Authors:** Boram Mok, Young Su Jang, Ji Hwan Moon, Sujin Moon, Yun Kyung Jang, Soo Yun Kim, Sung Joo Jang, Sang Hyun Moh, Dong Hyun Kim, Jung U Shin

**Affiliations:** 1 Department of Biomedical Science, CHA University School of Medicine, CHA University, Seongnam 13488, Republic of Korea; 2 Samsung Genome Institute, Samsung Medical Center, Seoul 06351, Republic of Korea; 3 Department of Dermatology, Bundang CHA Medical Center, School of Medicine, CHA University, Seongnam 13496, Republic of Korea; 4 Plant Cell Research Institute of BIO-FD&C Co., Ltd., Incheon 21990, Republic of Korea

**Keywords:** *Campanula takesimana*, callus, skin barrier, atopic dermatitis

## Abstract

Atopic dermatitis (AD) is a prevalent inflammatory skin disease characterized by epidermal barrier dysfunction and Th2-skewed inflammation. *Campanula takesimana* (*C. takesimana*), a Korean endemic plant grown on Ulleng Island, has long been associated with a traditional alternative medicine for asthma, tonsillitis, and sore throat. In this study, we reported the effect of *C. takesimana* callus extract on upregulating epidermal barrier-related proteins dysregulated by Th2 cytokines. *C. takesimana* callus extract induced the expression of skin barrier proteins, such as filaggrin, claudin-1, and zonula occludens-1, in both human primary keratinocytes and Th2-induced AD-like skin-equivalent models. Additionally, RNA sequencing analysis demonstrated that *C. takesimana* callus extract partially restored Th2 cytokine-induced dysregulation of the epidermal development and lipid metabolic pathways. Considering the advantages of callus as a sustainable eco-friendly source of bioactive substances, and its effect on skin barrier proteins and lipid metabolic pathways, *C. takesimana* callus extracts can possibly be utilized to improve the integrity of the skin barrier.

## 1. Introduction

A typical pathological feature of atopic dermatitis (AD) is skin barrier dysfunction. Filaggrin (FLG), a filament-associated protein that binds to keratin fibers in epithelial cells, plays an essential role in stratum corneum formation and barrier function [1,2,3,4]. During the final stage of epidermal development, FLG is broken down into free amino acids and derivatives, serving as a source of natural moisturizing factors (NMFs) that control stratum corneum hydration and pH [5,6,7,8]. Tight junctions in the granular layer support the formation of the skin barrier and regulate transdermal water loss [9]. Transmembrane proteins such as claudin-1 (CLDN-1) and zonula occludens-1 (ZO-1), which constitute tight junctions, have been reported to be reduced in the skin of patients with AD [9,10,11]. Th2 cytokines, such as IL-4 and IL-13, downregulate the expression of FLG and tight junction proteins, leading to the disruption of skin barrier function [2,12,13]. Defects in skin barrier proteins contribute to dry and inflamed skin, providing a site for allergen penetration and sensitization [14,15,16,17]. Additionally, damaged epithelial cells secrete chemokines and pro-inflammatory cytokines, such as interleukin IL-1, thymic stromal lymphopoietin, IL-25, and IL-33, further promoting Th2-skewed inflammation [18,19]. Therefore, effectively restoring the Th2-induced dysregulation of skin barrier proteins can improve skin barrier function in AD patients.

*Campanula takesimana* (*C. takesimana*), also known as Korean bellflower or Seomchorongkkot, is a Korean endemic herb that grows on Ulleng Island. Ulleng Island is a volcanic island located in the East Sea of Korea, characterized by various plant communities. This island exhibits unique vegetation and plant distribution influenced by environmental characteristics [20]. Currently, there are 550 species of native plants, including *C. takesimana*. In Korean folklore, *C. takesimana* has been associated with an effective herbal remedy for Th2-mediated diseases, such as bronchitis and asthma [21]. Furthermore, the root of *C. takesimana* has been used in herbal medicine to treat asthma, tonsillitis, and sore throat [20]. However, the mechanisms by which *C. takesimana* regulates Th2 inflammatory diseases are not well understood.

A callus is defined as a mass of dedifferentiated cells which possesses totipotency [22]. Calluses exhibit high plasticity for cell differentiation, which is a central characteristic. The induction of a callus in vitro has significant benefits compared to using the plant itself as a source of ingredients with biological activity, as it does not harm the environment and can be grown in vitro.

In this study, we induced calluses from *C. takesimana* and validated their efficacy on the expression of barrier proteins in primary keratinocytes and 3D skin equivalents. The extract from a *C. takesimana* callus successfully restored the Th2-induced dysregulation of the skin barrier-associated proteins and lipid metabolic pathways. These findings suggest that *C. takesimana*, a native herb of Ulleng Island, may be useful in enhancing epidermal barrier function.

## 2. Results

### 2.1. Induction of Calluses and the HPLC Analysis of the Callus Extract

**Figure 1:**
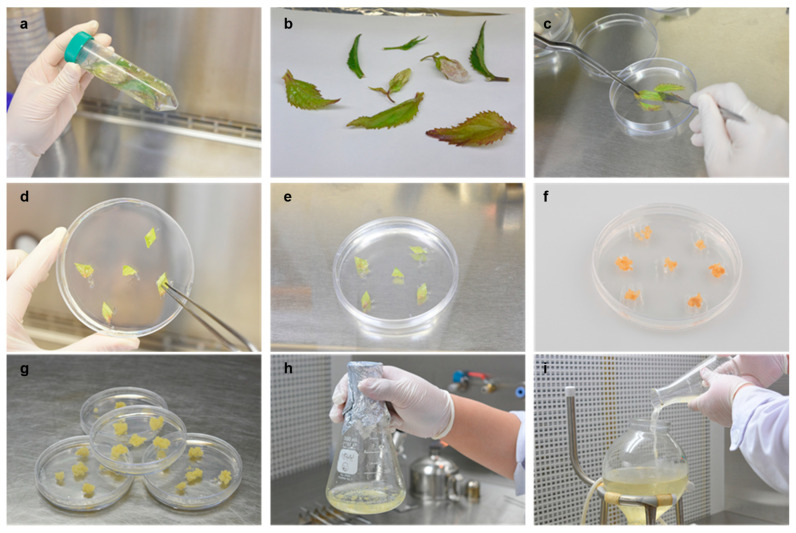
Callus induction from *Campanula takesimana* (*C. takesimana*). (**a**,**b**) Sterilization of leaf tissue. (**c**) Cutting the leaf tissue for plating. (**d**,**e**) Plating the leaf tissue on callus induction media. (**f**) Callus formation out of the leaf tissue. (**g**) Selecting a callus with the highest growth rate with no browning. (**h**) Suspension culture. (**i**) Bioreactor culture for mass production.

We induced callus formation from *C. takesimana* leaves using callus culture technology. The sterilized and cut *C. takesimana* leaves were placed in gel media supplemented with various combinations of auxin and plant growth regulators (PGRs) (Figure 1a–c). After 4 weeks, undifferentiated calluses had formed (Figure 1d,e), with several calluses turning brown by 8 weeks (Figure 1f). We selected the culture conditions that promoted the highest growth rate with minimal browning of the callus and propagated it in the chosen culture medium supplemented with 1 mg/L of 2,4-dichlorophenoxyacetic acid, an auxin-based growth regulator. Calluses grown in the selected culture medium exhibited no browning (Figure 1g). Subsequently, we transferred the calluses from the gel medium to liquid medium supplemented with 1 mg/L of 2,4-dichlorophenoxyacetic acid and cultured them in 10 L-capacity bioreactors for mass culture of the calluses (Figure 1h,i). The calluses were harvested and dried to extract bioactive compounds.

Next, we analyzed the *C. takesimana* callus extract using high-performance liquid chromatography (HPLC) and compared it to those in the *C. takesimana* plant extract. The analysis of both the plant extract and the callus extract revealed differences in their components, as observed in the chromatograms. In the callus extract, guanine, adenine, uridine, adenosine, and guanosine were detected, whereas these peaks did not appear in the plant extract (Figure S1a,b), indicating the higher proliferating activity of the callus compared to the plant itself.

### 2.2. Decreased Expression of FLG, ZO-1, and CLDN-1 by Th2 Cytokines Was Reversed by the C. takesimana Callus Extract

To explore the function of the *C. takesimana* callus extract on the skin, we assessed its impact on IL-4/IL-13-induced alterations in normal human epidermal keratinocytes (NHEKs). Various concentrations of the *C. takesimana* callus extract were added to the NHEK culture medium for 24 h for the viability and proliferation assay. Within the concentration range of 0–4 mg/mL, the callus extract significantly increased keratinocyte viability (Figure S2a) and proliferation (Figure S2b) at a concentration of 0.4 mg/mL. Based on the results, we selected concentrations of 0.04 mg/mL and 0.4 mg/mL for further experiments.

**Figure 2:**
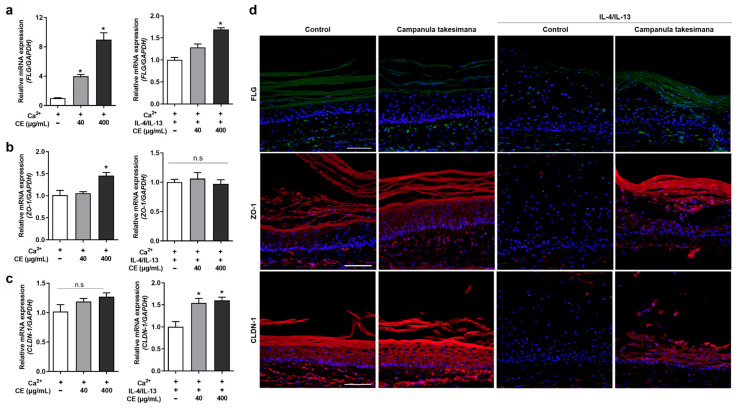
*C. takesimana* callus extract induces FLG, ZO-1, and CLDN-1 expression in NHEKs and skin equivalents. (**a**–**c**) A total of 40 µg/mL or 400 µg/mL of *C. takesimana* callus extract in the absence (**left** panel) or presence (**right** panel) of 10 ng/mL of IL-4/IL-13. The mRNA expression for (**a**) *FLG*, (**b**) *ZO-1*, and (**c**) *CLDN-1* was measured via qRT-PCR. (**d**) Skin equivalents were treated with *C. takesimana* callus extract with or without 100 ng/mL of IL-4/IL-13. The expression of FLG, ZO-1, and CLDN-1 was detected via immunofluorescence staining. Hoechst was used for nuclear staining. Scale bar = 50 μm. All data were analyzed using the one-way ANOVA followed by Tukey’s multiple comparisons test and shown as the mean ± SD (*n* = 4 per group; * *p* < 0.05; n.s. not significant).

Barrier-associated proteins such as FLG and tight junction proteins, including ZO-1 and CLDN-1, are known to be downregulated in the skin of individuals with AD due to enhanced Th2 inflammation [23,24,25]. To mimic Th2-induced barrier disruption, we treated NHEKs with Th2 cytokines, namely IL-4 and IL-13. The mRNA expression of FLG increased in calcium chloride-treated differentiated keratinocytes, but was significantly decreased by IL-4 and IL-13 treatment (Figure S3a,b). Next, we investigated whether treatment with the *C. takesimana* plant extract or *C. takesimana* callus extract on differentiated keratinocytes could regulate the expression of epidermal barrier-associated proteins. Although the *C. takesimana* plant extract had no effect on FLG expression (Figure S4a,b), the *C. takesimana* callus extract induced FLG expression in a concentration-dependent manner in differentiated keratinocytes, both in the absence of Th2 cytokines (Figure 2a, left panel) and in their presence (Figure 2a, right panel).

When we treated differentiated NHEKs with the *C. takesimana* callus extract, the mRNA expression of the tight junction protein ZO-1 increased at 400 µg/mL (Figure 2b, left panel). However, in the presence of IL-4 and IL-13, the callus extract did not significantly alter ZO-1 expression (Figure 2b, right panel). On the other hand, CLDN-1 expression was unaffected by the callus extract in differentiated primary keratinocytes without Th2 cytokines (Figure 2c, left panel), but it was significantly elevated by the callus extract in the presence of IL-4 and IL-13 (Figure 2c, right panel).

To validate the impact of the callus extract on epidermal development, we used skin equivalents cultured with IL-4 and IL-13. IL-4 and IL-13 significantly downregulated FLG, ZO-1, and CLDN-1 expression in the epidermal layer of the skin equivalents, and the *C. takesimana* callus extract successfully restored the expression of these skin barrier-related proteins (Figure 2d). These findings collectively demonstrated that the callus extract from *C. takesimana* exhibits a beneficial effect on upregulating epidermal barrier proteins and tight junction proteins that are downregulated in AD-affected skin.

### 2.3. The C. takesimana Callus Extract Restores Lipid Biosynthesis and the Epidermal Development Pathway

**Figure 3:**
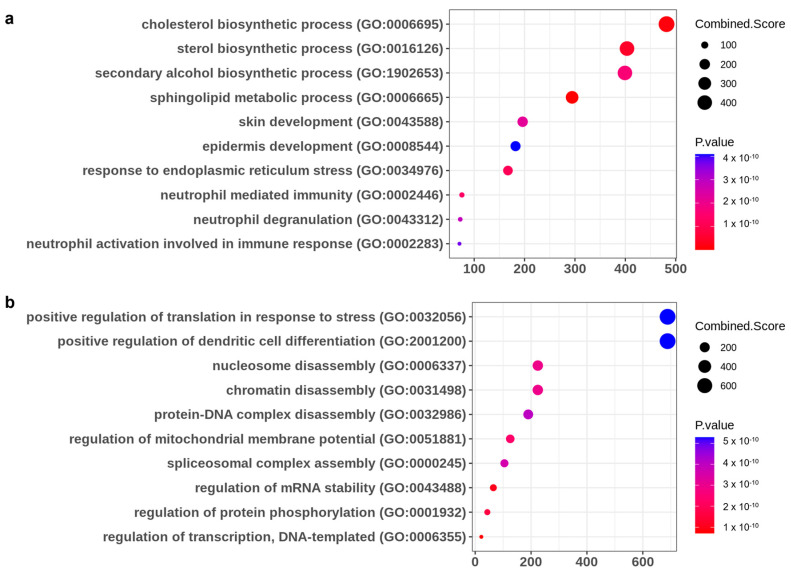
Gene set enrichment analysis comparing DEGs in control NHEKs, IL−4/IL−13-treated NHEKs, and NHEKs treated with both IL−4/IL−13 and *C. takesimana* callus extract. (**a**) The top 10 gene ontology terms enriched in genes downregulated by Th2 cytokines and upregulated by the *C. takesimana* callus extract. (**b**) The top 10 gene ontology terms enriched in genes upregulated by Th2 cytokines and downregulated by the *C. takesimana* callus extract.

We analyzed the whole genes that are downregulated or upregulated by Th2 cytokines and restored with the *C. takesimana* callus extract in differentiated NHEKs via RNA sequencing analysis. A total of 609 differentially expressed genes (DEGs) were identified as downregulated by Th2 cytokines and upregulated by the *C. takesimana* callus extract. On the other hand, 148 DEGs were identified as upregulated by Th2 cytokines and downregulated by the *C. takesimana* callus extract. The top 30 upregulated and downregulated DEGs by the *C. takesimana* callus extract are demonstrated in Table 1. Gene ontology (GO) analysis demonstrated that several pathways were restored with the *C. takesimana* callus extract, which was suppressed by Th2 cytokines. These pathways include the cholesterol biosynthetic process, sterol biosynthetic process, sphingolipid metabolic process, epidermis development, and response to endoplasmic reticulum stress (Figure 3a). Regarding the GO pathways that were increased by Th2 cytokines and decreased by the *C. takesimana* callus extract, they include the positive regulation of translation in response to stress, nucleosome disassembly, and regulation of mitochondrial membrane potential, among others (Figure 3b).

**Figure 4:**
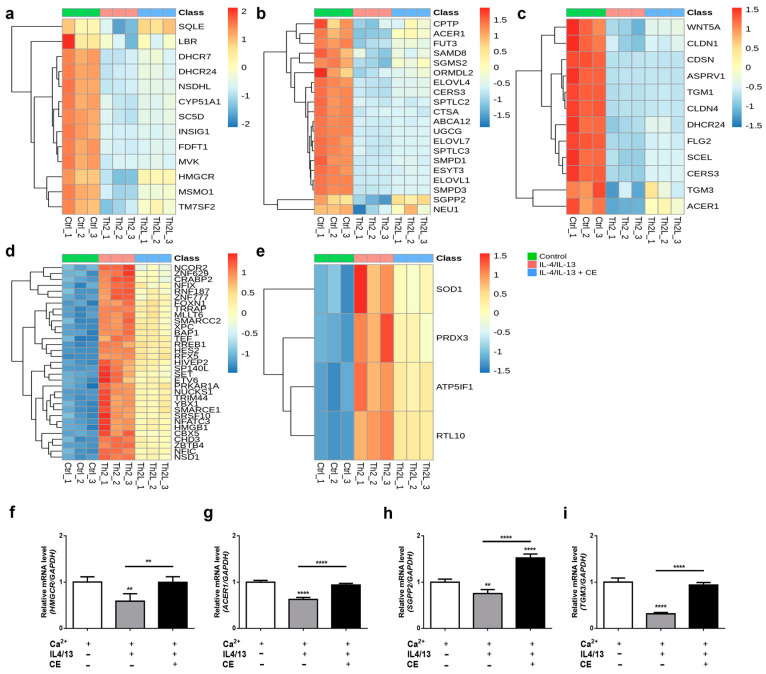
The effect of the *C. takesimana* callus extract on the DEGs reduced by Th2 cytokines. (**a**) The cholesterol biosynthesis pathway. (**b**) Sphingolipid metabolic processes. (**c**) Skin development. (**d**) Regulation of transcription (DNA templated). (**e**) Regulation of the mitochondrial membrane potential (*n* = 3 per group; green=control, red=IL4/IL13, blue= IL4/IL13 + *C. takesimana* callus extract). The mRNA expression of (**f**) *HMGCR*, (**g**) *ACER1*, (**h**) *SGPP2*, and (**i**) *TGM3* was analyzed via qRT-PCR. All data were analyzed via the one-way ANOVA analysis followed by Tukey’s multiple comparisons test and shown as the mean ± SD (*n* = 4 per group; ** *p* < 0.01 and **** *p* < 0.0001).

Several genes related to cholesterol biosynthesis, including squalene epoxidase (SQLE), 3-hydroxy-3-methylglutaryl-CoA reductase (HMGCR), methylsterol monooxygenase 1 (MSMO1), and transmembrane 7 superfamily member 2 (TM7SF2), were significantly reduced by IL-4 and IL-13 but were restored with the *C. takesimana* callus extract (Figure 4a). Similarly, genes involved in sphingolipid metabolic processes, such as sphingosine-1-phosphate phosphatase 2 (SGPP2), neuraminidase 1 (NEU1), and ceramide-1-phosphate transfer protein (CPTP), exhibited the same pattern (Figure 4b). Additionally, various genes associated with skin development, including the tight junction protein CLDN1 and the keratinocyte differentiation-related protein FLG2, were markedly reduced by Th2 cytokines and partially restored with the *C. takesimana* callus extract. Among them, the expression of alkaline ceramidase 1 (ACER1), which plays a significant role in maintaining the barrier function of the skin, was markedly restored with the *C. takesimana* callus extract (Figure 4c). Several genes involved in the regulation of transcription, DNA templated, were highly upregulated in the presence of IL-4 and IL-13. However, their relative expression levels were significantly reduced by the *C. takesimana* callus extract (Figure 4d). Likewise, genes involved in the regulation of mitochondrial membrane potentials, such as superoxide dismutase 1 (SOD1) and peroxiredoxin 3 (PRDX3), displayed a similar trend (Figure 4e).

To validate the RNA sequencing analysis, we confirmed the expression of HMGCR, ACER1, SGPP2, and TGM3, which are involved in the cholesterol biosynthesis pathway, sphingolipid metabolic processes, and skin development, through qRT-PCR. Consistent with the RNA sequencing results, these genes were downregulated in Th2 cytokine-treated keratinocytes and restored in callus extract-treated keratinocytes (Figure 4f–i).

## 3. Discussion

Previous studies have provided evidence for the antioxidative and anti-inflammatory effects of *C. takesimana* [20,21]. The ethyl acetate fraction of the *C. takesimana* extract exhibited free radical scavenging activity comparable to that of ascorbic acid [20]. Moreover, the extract of *C. takesimana* leaves has been found to inhibit the production of prostaglandin E2 induced by lipopolysaccharides [26]. These previous findings suggest the potential therapeutic value of *C. takesimana* in modulating inflammatory responses. In our study, we found another beneficial effect of *C. takesimana* in that the callus of *C. takesimana* increases the expression of barrier-related proteins and potentially restores lipid metabolism and the epidermal development process.

In this study, we induced calluses from the leaves of *C. takesimana* and obtained an extract using hot water. Since *C. takesimana* has been used as an herbal medicine to improve bronchitis and asthma in Korea, we attempted to investigate whether *C. takesimana* can restore barrier-related proteins affected by Th2 cytokines. We observed that the *C. takesimana* callus extract not only increased the expression of FLG, ZO-1, and CLDN-1 in the keratinocytes but also in the 3D skin equivalents. Interestingly, these effects of *C. takesimana* on FLG expression were only observed in the callus extract. When the callus extract and the plant extract were treated with the differentiating NHEKs, the plant extract did not induce FLG expression and did not restore the reduced FLG expression caused by Th2 cytokines.

Th2-skewed immune response is a key pathomechanism in AD. Increased Th2 cytokines, including of IL-4 and IL-13, lead to a reduction in skin barrier-related proteins, such as FLG, and impair skin barrier function. Several studies have shown that a lack of FLG interferes with epidermal maturation function, as well as alters skin lipid composition and organization. In addition, FLG mutations cause reduced NMF, which increases skin pH and accelerates barrier dysfunction [4,5,6]. Furthermore, tight junction proteins expressed in the stratum granulosum are another core component in the formation of the epidermal barrier. Tight junctions and the stratum corneum have a synergistic effect on the formation of a strong skin barrier, and reduced expression of tight junction proteins and decreased skin barrier function have been well reported in AD [27,28]. Thus, restoring skin barrier-related proteins is important in controlling the disease activity of AD. Since our study showed that the extract of *C. takesimana* callus successfully restored Th2-induced dysregulation of FLG and tight junction proteins, we speculate that it could improve the skin barrier function of patients with AD.

In addition to epidermal differentiation and barrier formation, RNA sequencing analysis revealed that lipid metabolism-related pathways, including the cholesterol biosynthetic process, sterol biosynthetic process, and sphingolipid metabolic process, were initially suppressed by Th2 cytokines but partially restored with the *C. takesimana* callus extract. In AD, there is a reduction in ceramides, one of the main lipids that surround the outer layer of corneocytes, within the stratum corneum [29,30]. Th2 cytokines can regulate the expression of ceramide metabolic enzymes and modulate ceramide levels [31,32]. The diminished synthesis of ceramides, which are important components of the skin barrier lipids, leads to impaired formation of the lamellar body, corneocyte lipid envelope, and an overall reduction in epidermal lipids, resulting in skin barrier dysfunction. In our study, IL-4 and IL-13 reduced the expression of genes associated with the lipid metabolic pathways, and the *C. takesimana* callus extract restored the expression of these genes. These results indicate that the *C. takesimana* callus extract can not only restore barrier protein expression but also restore the ceramide metabolic pathway.

Plant cells possess high plasticity for cell differentiation. In response to various stresses, such as wounding or pathogen infection, plants can generate unorganized cell masses known as calluses. A callus is defined as a mass of dedifferentiated cells or somatic embryos with a single-cell origin, meaning the callus is similar to plant cells in that it possesses totipotency. The induction of a callus in vitro has significant benefits compared to using the plant itself as a source of ingredients with biological activity, as it does not destroy the environment and can be grown in vitro. Additionally, the callus compared to the plant itself could be more biologically beneficial, as demonstrated in our study.

Taken together, the *C. takesimana* callus extract could help in regulating skin barrier proteins and lipid metabolism pathways, ultimately strengthening the skin barrier function. Considering the advantages of calluses as a sustainable and eco-friendly source of active materials, *C. takesimana* callus extracts could be possibly utilized for improving skin barrier function.

## 4. Materials and Methods

### 4.1. Induction of the Callus and Optimization of Culture Medium

To induce the callus, the *C. takesimana* leaves were sterilized sequentially with 70% ethanol for 30 s and 0.3% sodium hypochlorite for 20 min, washed with distilled water three times, and then cut into 0.5–1 cm portions. The leaves were cultured in a medium supplemented with various combinations of auxin and plant growth regulator (PGR) under dark conditions at 25 ± 2 °C. After 8 weeks, the color, morphology, and differentiation of the callus were compared, and the best combination of auxin and PGR for culture was selected. The *C. takesimana* callus was then mass-cultured in the selected culture medium.

### 4.2. Preparing Test Samples from C. takesimana Callus and Leaf

The callus was dried at 60 °C for 2 days to remove water and then powdered. The dried plant material and callus of *C. takesimana* were extracted under reflux by adding 25 times the volume of water and heating at 100 °C for 1 h. Each extract was centrifuged at 13,000 rpm for 10 min and filtered through a 0.45 μm membrane filter (PTFE, Advantec, Tokyo, Japan) to obtain each extract stock solution.

### 4.3. HPLC Analysis of the Samples

To compare the chromatographic data of the water extracts from the callus and leaf, instrumental analysis using HPLC (1260 Infinity II system, Agilent Technologies, Santa Clara, CA, USA) was carried out. All reagents using their mobile phase were of HPLC grade. Water and acetonitrile of Samchun Pure Chemicals (Republic of Korea) and trifluoroacetic acid of Alfa Aesar (Schiltigheim, France) were used. The analytical column was Shim-pack GIS C18 (5 μm, 4.6 × 250 mm; Shimadzu, Kyoto, Japan), and the eluent was a mixture of mobile phase A (water containing 0.1% trifluoroacetic acid) and B (acetonitrile containing 0.1% trifluoroacetic acid). The elution was performed under the following conditions: The elution was performed with mobile phase A at 100% (mobile phase B at 0%) for 5 min, followed by an increase in mobile phase B at a rate of 1% per minute. The flow rate was 1.0 mL/min (with an injection volume of 20 μL), and the data were collected at UV 255 nm. All samples were filtered through a 0.45 μm syringe filter (PTFE, Advantec) before injection.

### 4.4. Cell Culture

NHEKs were cultured in dermal cell basal medium (ATCC, Manassas, VA, USA) supplemented with bovine pituitary extract and recombinant growth factors (ATCC). Cultures were maintained in a 5% CO_2_, 37 °C incubator. For differentiation, cells were seeded and grown to 90% confluency and then cultured in the keratinocyte medium containing 1.5 mM CaCl_2_ for 5 days. Th2 cytokines, 10 ng/mL of IL-4 and IL-13 (Peprotech, NJ, USA), were treated with keratinocytes to induce the atopic dermatitis-like changes in gene expression in the presence or absence of the *C. takesimana* callus extract.

### 4.5. The Cell Viability and Proliferation Assay

Cell viability was measured using the EZ-Cytox Plus assay kit (Dogenbio, Seoul, Republic of Korea). Cells were seeded on 48-well plates at a density of 1.0 × 10^4^ cells/well, and the callus extract was treated at different concentrations for 24 h. The viability and proliferation assay was performed according to the manufacturer’s protocol. In brief, cells treated with the callus extract were exposed to 10 μL of EZ-Cytox reagent, followed by a 4 h incubation period. Subsequently, absorbance was measured at 480 nm using a microplate reader (Thermo Scientific, Waltham, MA, USA). Cell viability was calculated relative to the absorbance of the control group, and the cell proliferation rate was determined by dividing the absorbance by the protein quantity, normalized to the values of the control group.

### 4.6. RNA Isolation and Quantitative Real-Time PCR

The mRNA expression level was analyzed via quantitative real-time PCR using SYBR (Bioneer, Daejeon, Republic of Korea). Total RNA was isolated using TRIzol (Invitrogen, Waltham, MA, USA), and cDNA was synthesized using M-MLV reverse transcriptase (Promega, Madison, WI, USA) and oligo dT primers (Cosmogenetech, Seoul, Republic of Korea). The PCR conditions were as follows: initial denaturation for 15 min at 95 °C; followed by 40 cycles of 95 °C for 15 s, 60 °C for 45 s, and 72 °C for 30 s, for primer annealing and extension. Relative mRNA levels were calculated via normalization to the reference gene, GAPDH.

### 4.7. RNA Sequencing Data Analysis

RNA sequencing analysis was performed using differentiated NHEKs, which were treated with 10 ng/mL of IL-4 and 10 ng/mL of IL-13 with or without 0.4 mg/mL of callus extract for 5 days. RNA sequencing analyses were performed at Theragen Bio Institute (Suwon, Republic of Korea). The libraries were prepared for 150 bp paired-end sequencing using the TruSeq RNA sample prep kit (Illumina, San Diego, CA, USA). A total of 1 μg of RNA molecules was purified and fragmented, and then synthesized as single-stranded cDNAs via random hexamer priming. Using this as a template to synthesize the second strand, a double-stranded cDNA was prepared. cDNA libraries were amplified with PCR after a sequential process of end repair, A-tailing, and adapter ligation. The quality of these cDNA libraries was evaluated with the Agilent 2100 BioAnalyzer (Agilent, USA), and was quantified with the KAPA library quantification kit (Kapa Biosystems, Wilmington, MA, USA) in accordance with the manufacturer’s library quantification protocol. Cluster amplification of denatured templates was followed by paired-end (2 × 150 bp) sequencing using Illumina Novaseq6000 (Illumina).

FastQC (v0.12.0) was used to examine the quality of raw RNA-seq data, and the adapter sequences were removed using Trimmomatic (v0.40) [33]. Then, clean reads were aligned to the human reference genome (hg38) using STAR (v2.7.10a) [34], and RSEM (v1.3.3) [35] was used to quantify the gene expression levels. EBSeq [36] was used to calculate the differentially expressed genes (DEGs) based on the expected counts. The genes with a false discovery rate (FDR) of 0.05 or less and the absolute value of log2 fold change of 0.5 or higher were considered DEGs.

Gene set enrichment analysis (GSEA) was subsequently carried out on the DEGs to illustrate the functions of the *C. takesimana* callus extract and its biological pathways using Enrichr [37]. The gene ontology (GO) terms with a *p*-value less than 0.05 were considered statistically significant. The top 10 terms were analyzed and displayed using dot plots. The size of the dot represented the count of genes, and the color represents the *p*-value.

Heatmaps were generated to illustrate the effect of the *C. takesimana* callus extract by comparing the subsets of DEGs, which were selected based on having adjusted *p*-values of 0.05 or less and a log2 fold change value of 1.0 or higher. The relative intensities of the identified bioactive compounds were visualized using red color for the higher concentration and blue color for the lower concentration.

### 4.8. Producing a 3D-Reconstructed Human Skin Equivalent

The 3D-reconstructed human skin equivalents were made in triplicate, in accordance with the previous report [38]. Briefly, primary dermal fibroblast sheets were created by fibroblasts secreting their own extracellular matrix for 4 weeks. Primary keratinocytes were grown on a dermal sheet to form an epidermal layer. A dermal sheet and a dermal–epidermal equivalent were stacked to generate a full-thickness skin layer, and the skin equivalents were incubated at the air–liquid interface for 14 days. Ascorbic acid was added to the culture medium during the whole culture period to induce extracellular matrix secretion. The skin equivalents were grown in the culture medium with 100 ng/mL of IL-4 and IL-13 cytokines in the presence or absence of the callus extracts over a period of the air–liquid interface to make an in vitro atopic dermatitis-like 3D-reconstructed human skin equivalent model.

### 4.9. Immunofluorescence Staining

The 3D skin samples were fixed with 4% formaldehyde (Bio-solution, Suwon, Republic of Korea) overnight and embedded in OCT (Sakura Finetek, Tokyo, Japan). The 14 μm cryosections were fixed in ice-cold acetone for 20 min and incubated overnight at 4 °C with anti-ZO-1 (1:400, Abcam, Cambridge, MA, USA), anti-CLDN-1 (1:400, Abcam), and anti-FLG (1:200, Santa Cruz Biotechnology, Dallas, TX, USA) primary antibodies, followed by Alexa Fluor 488- or 594-conjugated secondary antibodies and DAPI (Invitrogen). All stained images were made with a digital camera (DP74, Olympus, Tokyo, Japan) coupled to an optical microscope (BX53, Olympus).

### 4.10. Statistical Analysis

All statistical analyses were performed on raw data using GraphPad Prism 6.0 (GraphPad Software, San Diego, CA, USA). Comparison groups were analyzed with the unpaired Student’s *t*-test for parametric distributions. For multiple comparisons, one-way ANOVA followed by Tukey’s post-hoc test was performed. All data were presented as the mean ± SD.

## Figures and Tables

**Table 1:** The top 30 upregulated and downregulated DEGs. FDR, false discovery rate; and FC, fold change. The functions of the genes were described with reference to http://www.ncbi.nlm.nih.gov/gene (accessed on 21 November 2023).

Gene	FDR	FC	Function of the Gene
Upregulated			
ARMC10	0	1.92075265	Direct interaction with the DNA-binding domain of p53 may play a role in cell growth and survival
ADIPOR1	0	1.80296869	Activation of an AMP-activated kinase signaling pathway, which affects levels of fatty acid oxidation and insulin sensitivity
VSIG8	0	1.6961644	Enables RNA-binding activity
AQP9	0	1.64439391	Allows the passage of a broad range of non-charged solutes
LIPK	0	1.63993719	Cornification
LIPM	0	1.63287672	Cornification
GPR87	0	1.62555006	G protein-coupled receptor
LIPN	0	1.61028427	Lipase that is highly expressed in granular keratinocytes
GALNT1	0	1.58661081	A member of the UDP-N-acetyl-alpha-D-galactosamine:polypeptide N-acetylgalactosaminyltransferase (GalNAc-T) family of enzymes
SLPI	0	1.58600084	Secreted inhibitor that protects epithelial tissues from serine proteases
BPIFC	0	1.5751966	Lipid-binding activity
YIF1A	0	1.57078565	Endoplasmic reticulum to Golgi vesicle-mediated transport
ERLEC1	0	1.5590954	Endoplasmic reticulum-associated degradation
CNPY2	0	1.55241235	Active in the endoplasmic reticulum
RHBDL2	0	1.53925667	Release soluble growth factors via proteolytic cleavage
EI24	0	1.52976924	A putative tumor suppressor
GJB6	0	1.50980776	Transport of ions and metabolites between the adjacent cells
SGMS2	0	1.4972011	Transfer of phosphocholine from phosphatidylcholine onto ceramide
NFE2L3	0	1.49358595	Heterodimerizes with small musculoaponeurotic fibrosarcoma factors to bind antioxidant response elements in target genes.
PI3	0	1.48234845	Elastase-specific inhibitor that functions as an antimicrobial peptide against Gram-positive and Gram-negative bacteria, and fungal pathogens
KRTDAP	0	1.48098994	Regulation of keratinocyte differentiation and maintenance of stratified epithelia
ANKRD22	0	1.4777997	Unknown
AADACL2	0	1.47697589	Enable hydrolase activity.
RDH12	0	1.47588748	NADPH-dependent retinal reductase
NLRP10	0	1.47438664	Regulatory role in the innate immune system
GORASP2	0	1.47282828	Stacking of Golgi cisternae and Golgi ribbon formation, as well as Golgi fragmentation during apoptosis or mitosis
ADIPOR2	0	1.47198093	Mediate increased AMPK and PPAR-alpha ligand activities, as well as fatty acid oxidation and glucose uptake by adiponectin
MUC15	0	1.47072629	Located in the Golgi lumen and plasma membrane
ELOVL4	0	1.47011945	Biosynthesis of fatty acids
TNFAIP6	0	1.45982512	A secretory protein that contains a hyaluronan-binding domain
Downregulated			
DAZAP2	0	0.78833111	A proline-rich protein which interacts with the deleted in azoospermia (DAZ) and transforming growth factor-beta signaling molecule Smad anchor for receptor activation (SARA)
NCOR2	0	0.78563125	A member of a family of thyroid hormone- and retinoic acid receptor-associated co-repressors
CNBP	0	0.7824024	Functions in Cap-independent translation of ornithine decarboxylase mRNA and sterol-mediated transcriptional regulation
PDLIM1	0	0.77357901	Adapter to bring other LIM-interacting proteins to the cytoskeleton
PEBP1	0	0.74116878	Modulate multiple signaling pathways, including the MAP kinase (MAPK), NF-kappa B, and glycogen synthase kinase-3 (GSK-3) signaling pathways.
UBE2L3	0	0.7370626	Ubiquitination of p53, c-Fos, and the NF-kB precursor p105
S100A11	0	0.73682603	A member of the S100 family of proteins containing two EF-hand calcium-binding motifs; may function in motility, invasion, and tubulin polymerization
CFL1	0	0.73597491	Widely distributed intracellular actin-modulating protein that binds and depolymerizes filamentous F-actin and inhibits the polymerization of monomeric G-actin in a pH-dependent manner
NUDC	0	0.73283037	Spindle formation during mitosis and in microtubule organization during cytokinesis
SPRR1B	0	0.68475142	Crosslinked to membrane proteins by transglutaminase, forming an insoluble layer under the plasma membrane
SH3BGRL3	0	0.65155543	Located in nuclear bodies
NUCKS1	7.7716 × 10^−16^	0.74810539	Phosphorylated in vivo by Cdk1 during mitosis of the cell cycle
LITAF	1.3878 × 10^−14^	0.79073672	A DNA-binding protein; mediates the expression of TNF-alpha by directly binding to the promoter region of the TNF-alpha gene
MRFAP1	2.5513 × 10^−13^	0.77753748	An intracellular protein that interacts with members of the MORF4/MRG (mortality factor on chromosome 4/MORF4-related gene) family and the tumor suppressor Rb (retinoblastoma protein.)
SAP18	4.3332 × 10^−13^	0.6983495	A component of the histone deacetylase complex
LCE3D	9.0949 × 10^−13^	0.79201422	Keratinization
MTPN	1.2578 × 10^−12^	0.78751906	Encode both myotrophin and leucine zipper protein 6
SPRR2E	2.0592 × 10^−12^	0.78798856	A family of small proline-rich proteins clustered in the epidermal differentiation complex on chromosome 1q21
SUMO1	2.5585 × 10^−12^	0.6757033	Nuclear transport, transcriptional regulation, apoptosis, and protein stability
ARPC5	2.8555 × 10^−12^	0.79739882	One of seven subunits of the human Arp2/3 protein complex
DBI	8.7995 × 10^−12^	0.75534482	Lipid metabolism and the displacement of beta-carbolines and benzodiazepines
SH3BP4	3.2123 × 10^−11^	0.7996902	Cargo-specific control of clathrin-mediated endocytosis, specifically controlling the internalization of a specific protein receptor
IMPACT	1.1463 × 10^−10^	0.7043169	Actin-binding activity and ribosome-binding activity
PPARA	2.1865 × 10^−10^	0.72814971	DNA-binding transcription factor activity; RNA polymerase II cis-regulatory region sequence-specific DNA-binding activity; and lipid-binding activity
CNN2	7.2918 × 10^−10^	0.7927439	Structural organization of actin filaments
MYG1	1.3996 × 10^−9^	0.35205611	Nuclease activity
ZNF592	1.6664 × 10^−9^	0.78021028	Developmental pathway, and the regulation of genes involved in cerebellar development
CDKN1A	2.7186 × 10^−9^	0.79306698	Inhibits the activity of cyclin/cyclin-dependent kinase 2 or /cyclin-dependent kinase 4 complexes
TBC1D16	1.4699 × 10^−8^	0.78832422	Regulation of receptor recycling
PLAGL2	3.0912 × 10^−8^	0.78914576	A zinc-finger protein that recognizes DNA and/or RNA

## Data Availability

All related data are within the manuscript.

## Supplementary Materials

The following supporting information can be downloaded at: https://www.mdpi.com/article/10.3390/ijms242417333/s1.
