# Supplementary material for: The Potential of Campanula takesimana Callus Extract to Enhance Skin Barrier Function
Source: Int J Mol Sci. 2023 Dec 11;24(24):17333. doi: 10.3390/ijms242417333 (PMC10743976; doi:10.3390/ijms242417333)
Supplement: Supplementary file 1 [file ijms-24-17333-s001.zip › ijms-2722228-supplementary.pdf]

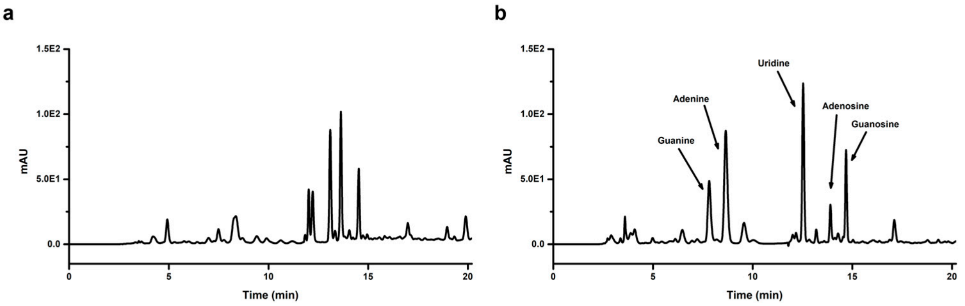

Figure S1. HPLC chromatograms of the *C. takesimana* leaf and callus extract
(a) *C. takesimana* leaf extract. (b) *C. takesimana* callus extract.

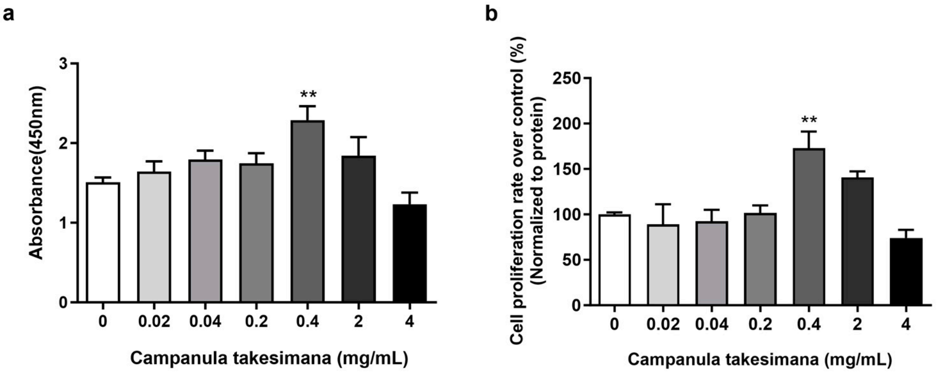

Figure S2. The effect of *C. takesimana* callus extract on cell viability and proliferation
(a) Absorbance of NHEKs treated with callus extract (0-4 mg/mL) was measured using MTT assay. (b) Cell proliferation rate was analyzed using EZ-Cytox Plus assay kit. All data are shown as mean ± SD and analyzed by one-way ANOVA analysis followed by Tukey's multiple comparisons tests (n = 4 per group, $^{**}p < 0.01$). $^{*}p < 0.05$

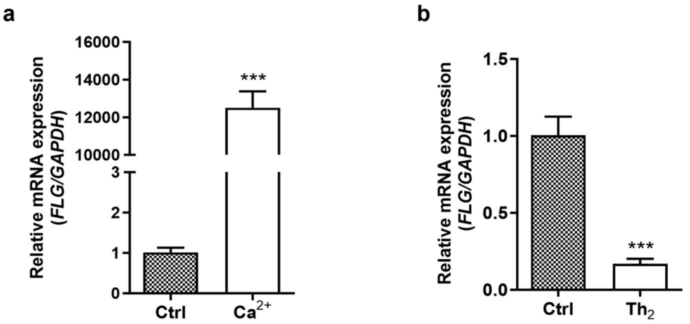

Figure S3. The effect of IL-4/IL-13 cytokines on *FLG* expression in NHEKs
(a, b) The mRNA expression of *FLG* was measured by qRT-PCR. (a) NHEKs were treated with $CaCl_2$ to induce the differentiation of the cells. (b) NHEKs were treated with $CaCl_2$ in the presence or absence of IL-4/IL-13 for 24h. All data are shown as mean ± SD and analyzed by an unpaired t-test (n = 4 per group, $^{***}p < 0.001$).

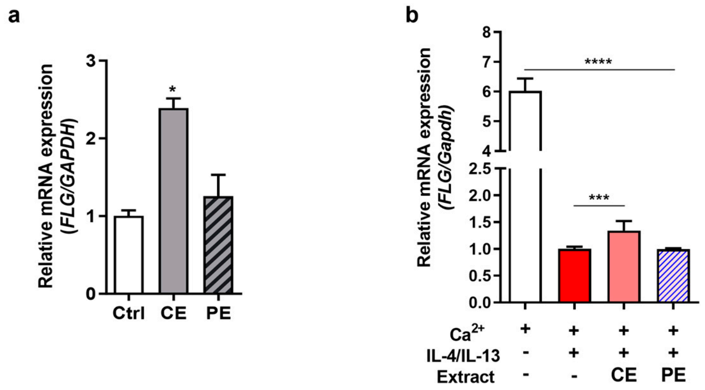

Figure S4. ***C. takesimana*** **callus extract, but not** ***C. takesimana*** **plant extract, regulates the expression of** ***FLG***

(a, b) Callus extract or plant extract was applied to the culture medium for 5 days with or without IL-4/IL-13 in the presence of calcium chloride. The expression of FLG was detected by qRT-PCR. All data were analyzed by one-way ANOVA analysis followed by Tukey's multiple comparisons test and shown as the mean ± SD (n=4 per group, *$p < 0.05$, **$p < 0.01$, ***$p < 0.001$, and ****$p < 0.0001$).
